# Epimedium Polysaccharide Ameliorates Benzene-Induced Aplastic Anemia in Mice

**DOI:** 10.1155/2020/5637507

**Published:** 2020-03-19

**Authors:** Jin He, Ru Han, Gongchang Yu, Martin F. Lavin, Qiang Jia, Ping Cui, Cheng Peng

**Affiliations:** ^1^Shandong Academy of Occupational Health and Occupational Medicine, Shandong First Medical University & Shandong Academy of Medical Sciences, Shandong, China; ^2^The University of Queensland Centre for Clinical Research, The University of Queensland, Brisbane, Queensland 4029, Australia; ^3^Queensland Alliance for Environmental Health Sciences (QAEHS), The University of Queensland, Brisbane, Queensland 4029, Australia

## Abstract

Benzene (BZ) is an important occupational and environmental pollutant. Exposure to BZ may cause aplastic anemia which is characterized as bone marrow hematopoietic failure. In order to reduce the harmful effects of this pollutant, it is necessary to identify additional preventative measures. In this study, we investigated the protective effects of epimedium polysaccharide (EPS), a natural compound with antioxidant and immune-enhancing potency, on aplastic anemia induced by benzene exposure in mice. Male CD-1 mice were randomly divided into five groups including control, BZ (880 mg/kg), LE (EPS low-dose, 20 mg/kg + BZ), ME (EPS middle-dose, 100 mg/kg + BZ), and HE (EPS high-dose, 200 mg/kg + BZ) groups. Animals were exposed to BZ by subcutaneous injection in the presence or absence of EPS via oral administration. All mice were treated 3 times a week for 8 consecutive weeks to develop a mouse model of benzene-induced aplastic anemia (BIAA). Results showed that BZ induced a significant decrease in both white and red blood cells, platelet counts, and hemoglobin level compared with that in the control group (*p* < 0.01). Treatment of EPS led to a protective effect against these changes particularly in the highest-dose group (HE, *p* < 0.01). EPS also recovered the decreased number of nucleated cells in peripheral blood cell smears and femur biopsies by BZ exposure. The increased level of reactive oxygen species (ROS) in bone marrow mononuclear cells (BMMNCs) in mice from the BZ group was significantly lower (*p* < 0.01) in the mice from the highest concentration of EPS (HE) group when compared with that from the control group. In addition, BZ exposure led to a significant increase in the apoptosis rate in BMMNCs which was prevented by EPS in a dose-dependent manner (*p* < 0.01). The antiapoptosis effect of EPS was through reversing apoptotic proteins such as BAX, Caspase-9 and Caspase-3, and Bcl-2. Finally, EPS treatment partially restored the levels of T cells and the different subtypes except CD80^+^ and CD86^+^ compared with the BZ group (HE, *p* < 0.05). These results suggest that EPS has protective effects against BIAA via antioxidative stress, immune modulation, and antiapoptosis mechanisms.

## 1. Introduction

Benzene (BZ) is an organic solvent that is widely used as a precursor in the synthesis of numerous products such as rubbers, dyes, insecticides, and drugs [1]. In addition, BZ is also an environmental chemical as it is used as an additive in gasoline and is present in cigarette smoke [2]. Studies have shown that chronic exposure to BZ can induce hematotoxicity and various blood diseases including aplastic anemia, myelodysplastic syndrome (MDS), and acute myeloid leukemia (AML) [1]. It is believed that BZ exerts its toxicity and carcinogenicity through its metabolites such as benzoquinone (BQ) and hydroquinone (HQ) [3–5]. These metabolites can accumulate in the bone marrow [6], where they cause oxidative impairment and apoptosis through the generation of reactive oxygen species (ROS) [7]. The bone marrow is not only a site for the storage of blood cells but also a key immune organ for adaptive and innate immune responses through stimulating lymphocytes [8, 9]. A recent epidemiological study suggested that immune-mediated chronic inflammation response is involved in BZ-induced hematotoxicity [10]. The T-cell-mediated immune response is associated with exogenous antigens which leads to a response in hematopoietic cells and immune cells to the particular situation resulting in inflammation [11]. BZ can cause bone marrow immunotoxicity partially through deregulating or suppressing T lymphocytes function [12] and cytokine production [13]. The immunological effects of BZ were demonstrated by a study showing the decreased levels of CD80^+^ T-cell and CD86^+^ T-cell expressions in peripheral blood lymphocytes of workers exposed to BZ [14]. In addition, clinical and animal experimental studies showed that BZ exposure also resulted in an immune suppression accompanied by a significant decrease of CD3^+^ and CD4^+^ T lymphocytes [15, 16]. These studies suggested that impairment of T-cell-mediated immune function plays an important role in BZ-induced toxicity. Therefore, immune modulation could be a potential target for protection against BZ-induced hematotoxicity [17].

Currently, there are no effective approaches to protect occupational workers from BZ-induced aplastic anemia (BIAA) and current treatment methods show limitations with short- and long-term side effects. Therefore, there is a need for new therapeutic approaches and the use of novel protective agents is required to prevent and treat the disease. Chinese herbal medicine has been widely used in China and in other Asian countries to treat various diseases and disorders. Epimedium polysaccharide (EPS) is the main effective constituent of Herba Epimedii (Ying Yang Huo in Chinese) and has been shown to have medicinal properties such as antioxidant, anti-inflammatory, and immune-modulatory effects *in vitro* and *in vivo* [18–21]. Polysaccharide has demonstrated its antioxidative effects through its free radical scavenging abilities [19]. The main constituents of EPS, such as flavonoids, have been shown to have the antioxidative ability and enhance the activity of the free radical scavenging enzyme in rat and chicken [19]. Pharmaceutical applications of EPS have been proposed based on its protective effects such as its antitumor activity and its ability to alleviate neurotoxicity [22, 23]. However, the therapeutic effects of EPS on BZ-induced hematotoxicity and immunotoxicity remain unclear. In this study, we established a mouse model of BIAA which has been developed successfully using different BZ exposure schemes [24, 25]. We treated them with various concentrations of EPS with the aim of assessing its potential protective effects against BZ-induced hematotoxicity and immunotoxicity in mice.

## 2. Material and Methods

### 2.1. Chemicals and Antibodies

Benzene (C_6_H_6_) with a purity of 99.7% was purchased from Merck (Germany, NO K8008T383540). EPS was purchased from Xi'an-Ciyuan (China, HPLC 98.15%). Wright-Giemsa stain, phosphate-buffered saline (PBS), and reactive oxygen species (ROS) assay kit (Cat. No. CA1410) were purchased from Solarbio (Shanghai, China). Annexin V-FITC/PI Apoptosis detection kit (Cat. No. AO2001-02P-G) was purchased from Sungene (Biotech, Tianjin, China). FITC labeled CD3^+^ (Catalog 100203), PE/Cy5 labeled CD4^+^ (Catalog 100410), PE/Cy7 labeled CD8^+^ (Catalog 100722), PE labeled CD80^+^ (Catalog 104707), and APC labeled CD86^+^ (Catalog 105011) antibodies were purchased from BioLegend (San Diego, American). Anti-B-cell-lymphoma-2 (Bcl-2) (Cat. No. 185002), anti-Bcl-2-associated X protein (BAX) (Cat. No. 18283), anti-Caspase-3 (Cat. No. 197202), anti-Caspase-9 (Cat. No. 219590), anti-*β*-actin, and horseradish peroxidase- (HRP-) conjugated secondary antibodies (IgG) were purchased from Abcam (SA, TX, USA) (Cat. No. 134988). All other chemicals with the analytical grade or the highest commercial grade available were from Beijing Chemical Works (Beijing, China).

### 2.2. Animals

Specific-pathogen-free male CD-1 mice, 8 weeks old, were purchased from Beijing Vital River Laboratory Animal Technology Co., Ltd. (Beijing, China). The mice were housed in an SPF Facility (temperature 23 ± 2°C, humidity range 40–70%, 12 h light/dark cycle). Food and UV-sterilized tap water were provided ad libitum. All procedures were conducted in strict accordance with the Guidelines for Animal Experiments published by the Ministry of Science and Technology of the People's Republic of China (GB14924.2-2001, China). Experiment procedures were approved by the Institutional Animal Care and Use Committee of Shandong Academy of Occupational Health and Occupational Medicine, Jinan, China.

### 2.3. Experimental Protocol

After one week of acclimatization, fifty CD-1 mice were divided into five groups randomly: control group, benzene group (BZ), low-dose EPS group (LE), medium-dose EPS group (ME), and high-dose EPS group (HE). BZ was dissolved in corn oil (BZ: corn oil = 1 : 1) and administered by subcutaneous injection according to 20 mL/kg body weight with a dose of 880 mg/kg. EPS was dissolved to 1 mg/mL in distilled water and gavage-administered at 10 mL/kg body weight with various concentrations (20, 100, and 200 mg/kg) 30 min prior to BZ subcutaneous injection. The EPS dosages were selected according to the results of a previous study [26] with some modifications. Mice from the control group received the equivalent volume of corn oil as the BZ vehicle by subcutaneous injection. All mice were treated 3 days a week for 8 consecutive weeks. The treatment scheme is listed in Table 1.

### 2.4. Evaluation of the BIAA Mouse Model

The BIAA mouse model was established according to the accepted criteria and evaluated by body weight, peripheral blood cell counts, peripheral blood cell smears, and femur biopsies [27]. The body weights of all mice were measured once a week using an automatic balance. To monitor the hematotoxicity and aplastic anemia during the exposure period, we collected approximately 20 *μ*L of blood from each mouse by tail vein puncture into EDTA-K2-containing tubes after 0, 10, 22, 33, 45, and 56 days of treatment. Counts of white blood cells (WBC)/leukocytes, red blood cells (RBC)/erythrocytes, platelets (PLT), and hemoglobin (Hb) concentration for each mouse were immediately measured using an automated hematology analyzer (Mindray BC-2800 Vet, China). Blood smears were also analyzed using an optical microscope (Olympus BX41, Olympus Corporation, Tokyo, Japan).

### 2.5. Isolation of Bone Marrow Mononuclear Cells (BMMNCs) and Histological Examination of Femur

After treatment completion with BZ and EPS for 8 weeks, all mice were sacrificed and BMMNCs were isolated from the right femur with saline buffer and suspended in red blood cell lysis buffer (Cat. No. R1010, Solarbio Science Technology, Beijing, China) to obtain BMMNCs. The BMMNCs were suspended in RPMI-1640 with 10% fetal bovine serum (FBS) and counted in a hemacytometer under an optical microscope. The left femurs of mice were fixed in 4% paraformaldehyde in 0.1 M PBS (pH 7.4), paraffin-embedded, sectioned, and H&E stained. The slides were then analyzed using an optical microscope (Olympus BX41, Olympus Corporation, Tokyo, Japan).

### 2.6. Measurement of ROS and Apoptosis in BMMNCs

For ROS detection, BMMNCs (1 × 106) were resuspended with 1 mL of 2,7-dichlorodi-hydrofluorescein diacetate (DCFH-DA) that was diluted in serum-free medium at a 1 : 1000 ratio and incubated at 37°C water bath for 30 min (mixed every 5 min). After 3 washes with PBS, the fluorescence intensity of the cells was analyzed using flow cytometry. BMMNCs were prepared in the same way as described in Section 2.5. The extent of apoptosis in freshly collected BMMNCs was analyzed by Annexin V-fluorescein isothiocyanate (FITC)/Propidium Iodide (PI) double staining. BMMNCs (1 × 106) were washed twice with cold PBS, resuspended in 300 *μ*L binding buffer with 5 *μ*L Annexin V/FITC, and incubated for 10 min at room temperature in the dark. Subsequently, 5 *μ*L of PI was added to the mixture and incubated for 5 min at room temperature in the dark. The sample was then analyzed using a flow cytometer (CytoFLEX, Beckman Coulter, USA).

### 2.7. Protein Analysis by Western Blot

BMMNCs were collected and lysed in RIPA buffer (Beyotime, Haimen, China) supplemented with protease and phosphatase inhibitors (Beyotime, Haimen, China) for 30 min. Protein concentrations were quantified using the BCA method. Proteins were electrophoresed on a 10 % SDS-PAGE gel, transferred onto polyvinylidene fluoride membranes, and then blocked with 5% nonfat milk for 1 h at room temperature. The membrane was then incubated with the following primary antibodies: rabbit anti-B-cell-lymphoma-2 (Bcl-2), rabbit anti-Bcl-2-associated X protein (BAX), rabbit anti-Caspase-3, rabbit anti-Caspase-9, and anti-*β*-actin overnight at 4°C. The membrane was subsequently washed three times for 5 min with Tris-buffered saline containing 0.1% Tween and incubated with HRP-conjugated secondary antibodies (IgG) for 60 min at room temperature. The blots were detected by chemiluminescence (Millipore, Billerica, USA).

### 2.8. Analysis of T Lymphocyte Subsets

Peripheral blood was collected from each mouse into EDTA-K2-containing tubes. 100 *μ*L of whole blood from each sample was added to individual test tubes containing preadded monoclonal antibodies against the following lymphoid cell surface markers: CD3, CD4, CD8, CD80, and CD86 and incubated for 30 min at room temperature in the dark. After adding 2 mL red blood cell lysis buffer, the solution was incubated for 15 min. Cells were pelleted at 300 g, washed 3 times with PBS, and resuspended in 200 *μ*L 1% formaldehyde. The cells were analyzed using a flow cytometer.

### 2.9. Statistical Analysis

The STATA 10.0 (http://www.stata.com/) software package was used to perform statistical analyses. Analysis of variance of the factorial design was performed for different treatment groups. The *t*-test was used to compare the different treatment groups. Statistical analysis was performed by one-way analysis of variance (ANOVA) and Turkey's multiple comparison posttest using GraphPad Prism 5 software (San Diego, CA, USA). Results were presented as means ± SD. *p* < 0.05 was considered statistically significant.

## 3. Results

### 3.1. Mice Exposed to BZ Showed Lower Body Weights

**Figure 1:**
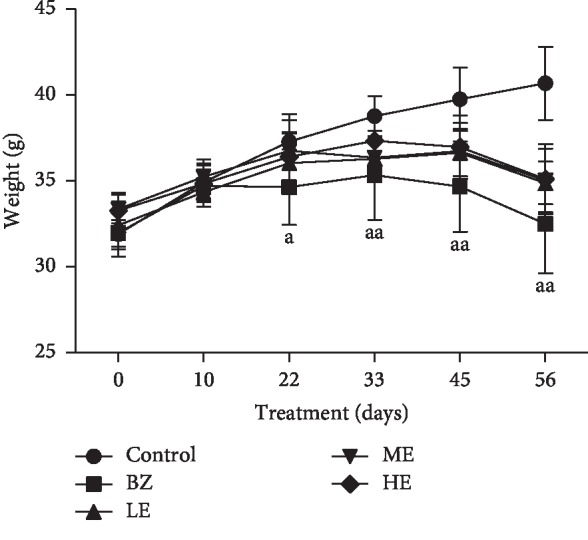
Body weight changes of mice treated with EPS and BZ for 8 weeks. Data was shown as mean ± SD (*n* = 10). Control: corn oil; BZ: benzene; LE: benzene + low-dose epimedium polysaccharide (20 mg/kg); ME: benzene + middle-dose epimedium polysaccharide (100 mg/kg); HE: benzene + high-dose epimedium polysaccharide (200 mg/kg). ^a^*p* < 0.05, ^aa^*p* < 0.01 indicates a significant difference compared with the control.

The body weights of mice from all the groups were summarized in Figure 1. After 8 weeks of BZ and EPS treatment, a gradual increase in the body weights was recorded for the control group (25%), BZ group (11%), HE group (17%), ME group (13%), and LE group (13%) as compared with their initial body weights. The lowest body weight was observed in the BZ group (11%). Overall, there was no statistically significant difference between the EPS treatment and control groups.

### 3.2. EPS Recovered the BZ-Induced Changes in Blood Parameters and Bone Marrow Cells Counts

**Figure 2:**
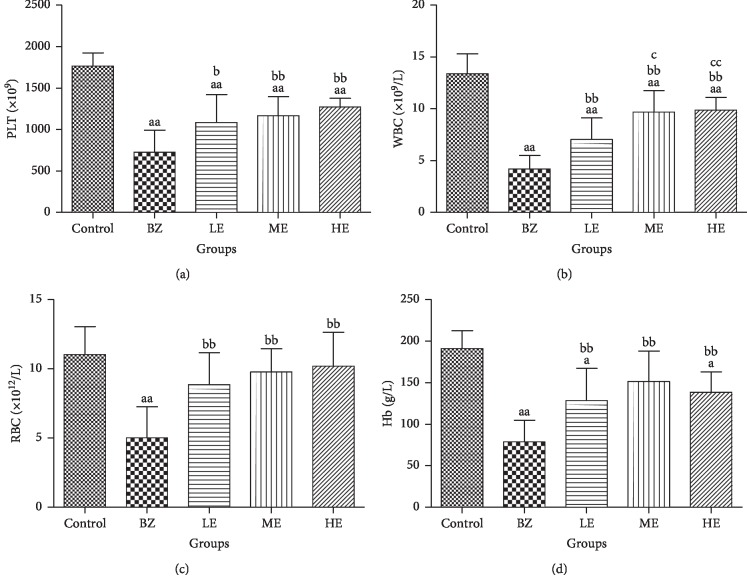
Effect of EPS and BZ on peripheral blood cell counts in mice. (a) PLT. (b) WBC. (c, d) RBC and Hb levels in control, BZ and LE, ME, and HE-treated groups. Data are shown as mean ± SD (*n* = 10). ^a^*p* < 0.05 and ^aa^*p* < 0.01 indicate a significant difference compared to the control. ^b^*p* < 0.05 and ^bb^*p* < 0.01 indicate a significant difference compared to the BZ group. ^c^*p* < 0.05 and ^cc^*p* < 0.01 indicate a significant difference compared to the LE group (20 mg/kg).

**Figure 3:**
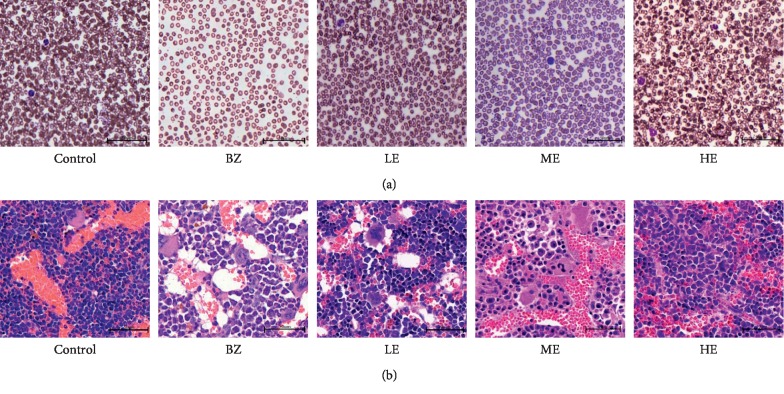
Effect of EPS and BZ on blood and bone marrow nucleated cells in mice. The nucleated cells were observed in peripheral blood smear (a) and femur biopsy (b) in mice of each group.

After treatment with BZ and EPS for 8 weeks, peripheral blood cell counts and Hb levels in mice from all the groups were analyzed. As shown in Figure 2, there was a significant decrease in WBC, RBC, PLT counts, and Hb levels in mice from the BZ group (*p* < 0.01) as compared to the control group. In contrast, EPS treatment significantly increased the counts of WBC, RBC, PLT (*p* < 0.01), and Hb level (*p* < 0.05) as compared to the BZ group. Furthermore, the HE group showed a higher count of WBC as compared to the LE and ME groups. H&E staining was also analyzed in blood smears and femur tissue to evaluate the potential effect of EPS on BZ-induced hematotoxicity. The results of peripheral blood and femur histology smear in mice from each group are shown in Figure 3. Compared to the control group, exposure to BZ significantly decreased the counts of blood cells in peripheral blood smears (Figure 3(a)), hematopoietic area, megakaryocytes, and hematopoietic cells and significantly increased nonhematopoietic cells (fat cells) in femur histology (Figure 3(b)). An upward trend in peripheral blood cells and bone marrow cells counts in both blood smears and femur histology with increasing EPS dosage (20, 100, and 200 mg/kg) was observed.

### 3.3. EPS Reduced the ROS Level of BMMNCs Exposed to BZ

**Figure 4:**
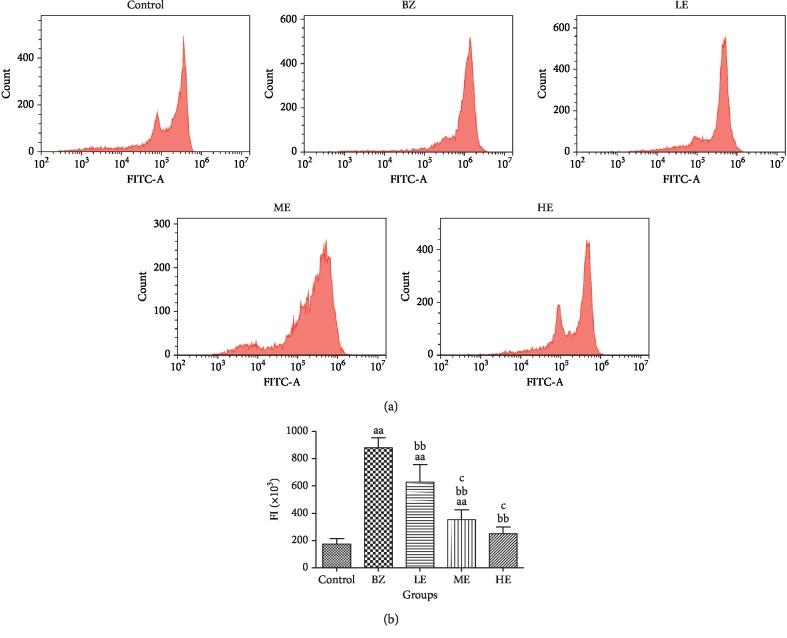
Effects of EPS and BZ on ROS production of BMMNC in mice. (a) ROS levels in BMMNCs of all groups. (b) Quantitation of the ROS levels by the FITC fluorescent values. Data are shown as mean ± SD (*n* = 10). ^a^*p* < 0.05 and ^aa^*p* < 0.01 indicate a significant difference compared to the control. ^b^*p* < 0.05 and ^bb^*p* < 0.01 indicate a significant difference compared to the BZ group. ^c^*p* < 0.05 indicates a significant difference compared to the LE group (20 mg/kg).

After treatment with BZ and EPS for 8 weeks, the level of ROS was measured in BMMNCs to investigate the antioxidant effect of EPS (Figure 4). The results showed that the level of ROS in BMMNCs significantly increased in the BZ group (*p* < 0.01) as compared to the control group. A significant decrease in the levels of ROS was detected in all EPS-treated groups as compared to the BZ group (*p* < 0.01). ROS levels were also lower in the ME and HE groups than those in the LE group (*p* < 0.01). These results indicated the dose-dependent antioxidant effect of EPS in BMMNCs from BIAA mice.

### 3.4. EPS Restored the Increased Apoptosis Rate of BMMNCs Exposed to BZ

**Figure 5:**
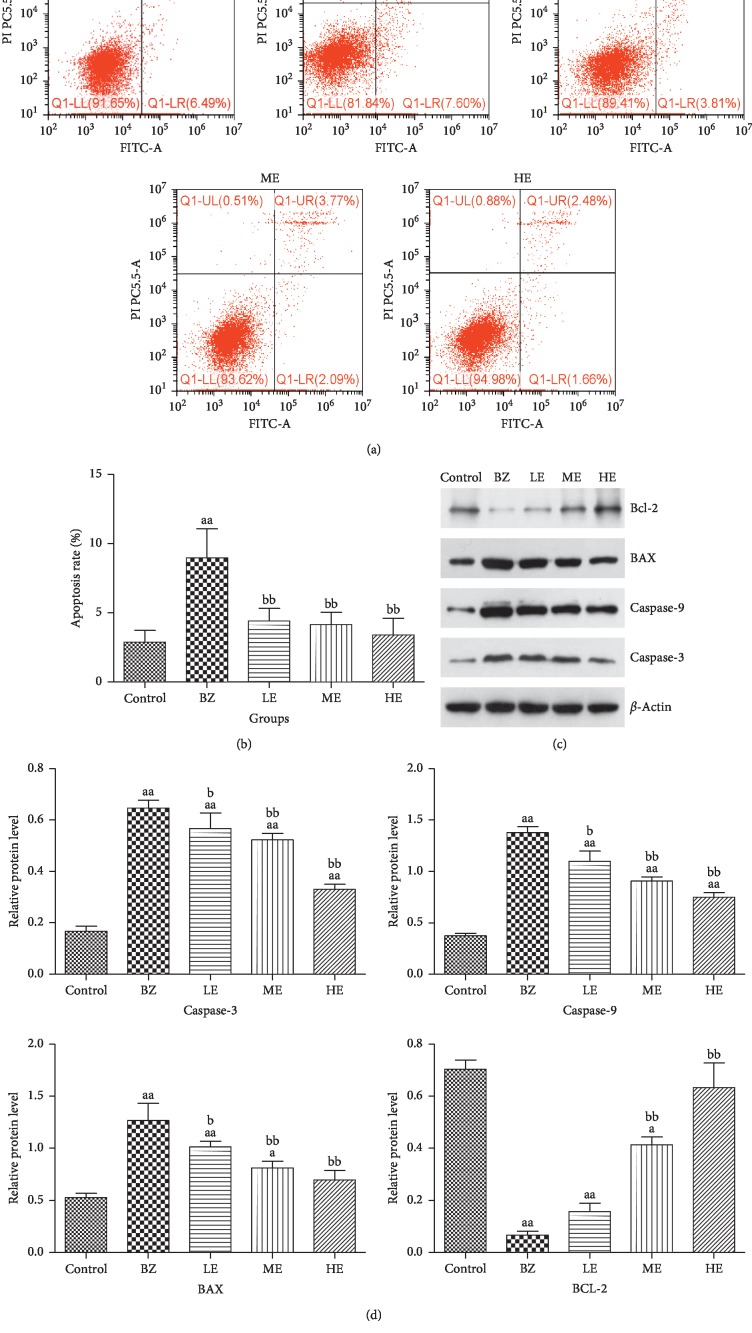
Effects of EPS and BZ on apoptosis amount and expression of the apoptosis-related protein in BMMNCs of all the mice. (a) Detection of apoptosis rate by flow cytometer (*n* = 10). (b) Comparison of the amount of apoptosis among all the groups. (c) Bcl-2, BAX, Caspase-9, Caspase-3, and *β*-actin expressions in BMMNCs of all the mice (*n* = 10). (d) Quantitation of expression levels of apoptosis-related protein. ^a^*p* < 0.05 and ^aa^*p* < 0.01 indicate a significant difference compared to the control. ^b^*p* < 0.05 and ^bb^*p* < 0.01 indicate a significant difference compared to the BZ group.

After completion of treatment with BZ and EPS for 8 weeks, the extent of apoptosis in BMMNCs in mice from all groups was determined. The results showed that there was a significant increase in the amount of apoptosis in the BMMNCs of the mice in the BZ group as compared to the control group (*p* < 0.01). In contrast, the extent of apoptosis in BMMNCs in the different EPS groups decreased significantly (*p* < 0.01) as compared to the BZ group. We also observed a decreasing trend of apoptosis in the BMMNCs of the mice with an increasing dosage of EPS (Figures 5(a) and 5(b)). Furthermore, the expression of proteins involved in apoptosis, namely, Bcl-2, BAX, Caspase-9, and Caspase-3 detected by immunoblotting revealed a significant increase in BAX, Caspase-9, and Caspase-3 with a decrease in Bcl-2 (*p* < 0.01) in the mice from BZ group as compared with those from the control group. Conversely, the relative protein expression levels of BAX, Caspase-9, and Caspase-3 were observed to be significantly decreased with an increase in Bcl-2 with increasing EPS dosage, particularly in the mice from HE group, as compared to BZ group. These differences were significant among the three EPS dosage groups (20, 100, and 200 mg/kg) (*p* < 0.05) (Figures 5(c) and 5(d)).

### 3.5. EPS Recovered T-Cell Numbers

**Figure 6:**
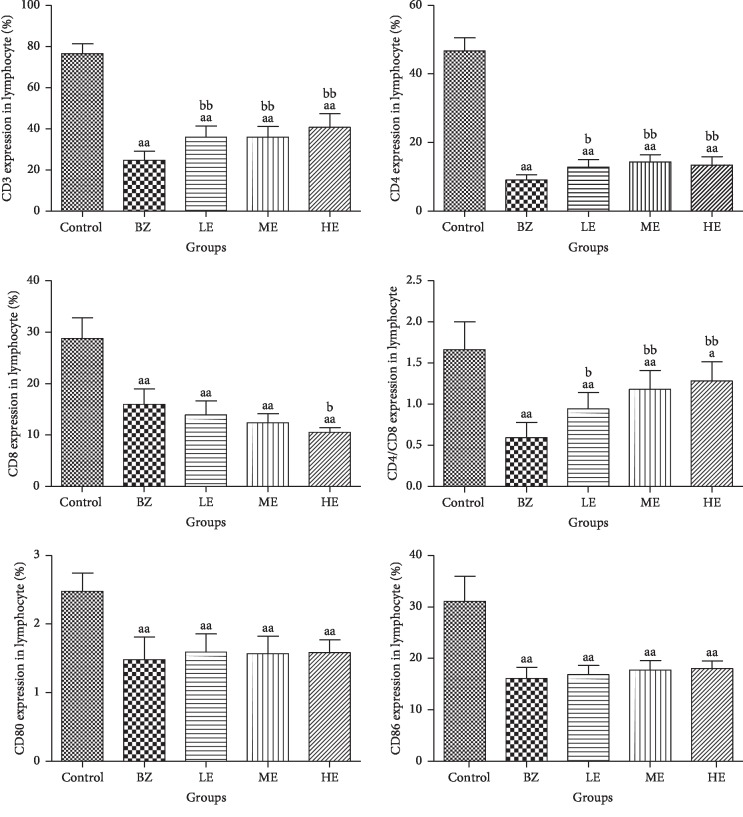
Effect of EPS and BZ on the percentage of CD3^+^, CD4^+^, CD8^+^, CD80^+^, and CD86^+^ T lymphocytes and CD4^+^/CD8^+^ ratio in peripheral blood of all the mice. Data are shown as mean ± SD (*n* = 10). ^a^*p* < 0.05 and ^aa^*p* < 0.01 indicate a significant difference compared to the control. ^b^*p* < 0.05 and ^bb^*p* < 0.01 indicate a significant difference compared to the BZ group.

Peripheral blood samples from mice in all groups were analyzed by flow cytometry in order to identify the changes in different T lymphocyte subsets present. As shown in Figure 6, the BZ group had a significant decrease in the percentage of CD3^+^, CD4^+^, CD8^+^, CD80^+^, and CD86^+^ T cells and the ratio of CD4^+^/CD8^+^ as compared to the control group (*p* < 0.01). However, the LE, ME, and HE groups had a significant recovery in the percentage of CD3^+^ and CD4^+^ T cells and CD4^+^/CD8^+^ ratio (*p* < 0.01) as compared to the BZ group, and the highest percentage of CD3^+^ T cells was observed in the HE group. The percentage of CD8^+^ T cells was significantly lower in the HE group than that in the BZ group (*p* < 0.01). There was no significant difference in the percentage of CD8^+^ T cells between the BZ group and the EPS groups except in the HE group (*p* < 0.05). The ratio of CD4^+^/CD8^+^ was observed to be significantly higher in the three EPS groups as compared to the BZ group, and the highest ratio of CD4^+^/CD8^+^ was observed in the HE group. An increasing trend of the percentage of CD80^+^ and CD86^+^ T cells was observed with the increasing dosage of EPS. However, no significant difference in the percentage of CD80^+^ and CD86^+^ T cells was observed between the EPS intervention groups and BZ group.

## 4. Discussion

BZ exposure was found to be one of the most common environmental stressors which induced excessive ROS production *in vivo* and *in vitro* [28, 29]. Although it is believed that the bone marrow is one of the main targets of BZ, the exact mechanisms underlying the hematotoxicity of BZ remain to be understood. Studies have shown that immunotoxicity and oxidative stress have been implicated in BZ-induced hematopoietic injury [10, 14, 28]. EPS is one of the active compounds of Chinese herbal medicine, Herba Epimedii, for treating cardiovascular diseases and improving sexual and neurological functions [19]. It has also shown efficacy in protecting against neurotoxicity via its antioxidative properties [23]. In this study, we investigated EPS by evaluating its effects on oxidative stress, apoptosis, and immunotoxicity induced by BZ in the BIAA mouse model.

Firstly, we treated CD-1 mice by subcutaneous administration of BZ at a dose of 880 mg/kg for 8 weeks to establish a BIAA mouse model successfully, a serious but treatable blood condition. The BIAA model was confirmed by a significant decrease in body weight and hematological parameters characteristic by pancytopenia as reported by a previous study [25]. Abnormalities such as anemia, leukocytopenia, and thrombocytopenia were also similar to those observed in patients with BZ-induced aplastic anemia [30, 31]. A loss of body weights was observed in mice treated with BZ by subcutaneous injection as shown in Figure 2, which suggested that the BZ has systemic effects and this is in agreement with the previous study [32]. However, EPS treatment alleviated the weight loss, which might be linked to the recovery of T-cell immune suppression. The reduced blood accounts in mice from the BZ group were likely due to bone marrow damage or injuries by BZ which was evidenced by the femur histological changes and a significant decrease in the hematopoietic area accompanied with significant increases of nonhematopoietic cells (fat cells) (Figure 3). However, neutrophils in the peripheral blood cells and hematopoietic cells including megakaryocytes in the bone marrow of mice from EPS-treated groups were significantly increased compared with those of BZ group, which suggested the protective effects of EPS on BIAA hematotoxicity by rescuing the counts of all blood cell types as well as bone marrow hematopoietic cells.

To explore the possible mechanisms involved in the protective effect of EPS, we investigated the toxicity of BZ and the effects of EPS at the molecular level. In the present study, mice treated with BZ showed a significant increase in ROS levels in BMMNCs which suggested that BZ-induced myelotoxicity is related to oxidative stress injury. This is expected since several studies confirmed the important role of oxidative stress in BZ-induced hematotoxicity [33, 34]. Metabolites of BZ such as hydroquinone (HQ) and benzoquinone (BQ) may be responsible for BZ-induced oxidative stress [13]. As expected, a higher level of ROS was observed in mice from the BZ group. After treatment with EPS, ROS levels were significantly reduced in comparison with those in mice treated with BZ only. The antioxidant property of EPS attributes to its hydroxyl radical scavenging activities, reducing power and stimulation effects on antioxidant enzyme activities such as SOD, Cat, and GSH-PX [21, 35]. The results indicated EPS can quench BZ-induced excessive ROS, which may play an important role in its protective effects on BZ-induced toxicity.

Accumulating evidence has indicated that oxidative stress plays an important role in the apoptosis signaling pathway [36–38]. ROS is an important trigger of DNA damage and induces cell apoptosis. In the present study, flow cytometry analysis confirmed the increased apoptosis in BMMNCs in our BIAA mice model. In order to reveal possible molecular mechanisms involved in apoptosis, we examined the expression of the apoptosis-related protein in BMMNCs using western blot. The Bcl-2 family proteins are the central regulators of apoptosis and have a critical role in the mitochondrion-mediated intrinsic pathway. Changes in the expression levels of Bcl-2, an antiapoptotic protein, and BAX, a proapoptotic protein, are often used as markers of apoptosis [39]. Caspase-dependent apoptosis is the classical programmed cell death pathway. Bcl-2 family protein activation may induce mitochondrial membrane changes and stimulate the release of cytochrome C. As ROS increases, cytochrome C is released which then triggers Caspase-3 and Caspase-9 activation, finally inducing a caspase signaling cascade and resulting in apoptosis [40]. *In vivo*, DNA damage can trigger the mitochondria-dependent Caspase-3 apoptosis pathway through ROS accumulation and changes in mitochondria membrane permeability [41]. Increased apoptosis of BMMNCs and/or CD34^+^ cells plays a major role in the pathogenesis of aplastic anemia patients [42]. Exposure to BQ, a metabolite of BZ, induces apoptosis in HL-60 cells through a ROS-dependent mechanism [4]. A study by Luo et al. has also indicated that HQ, another metabolite of BZ, may activate the intrinsic pathway of apoptosis via activation of Caspase-3 [43]. In the present study, flow cytometry analysis confirmed the increased apoptosis in BMMNCs in our BIAA mice model. In order to reveal a possible molecular mechanism that contributes to bone marrow suppression, we examined the expression of the apoptosis-related protein in BMMNCs. BZ exposure upregulated BAX, Caspase-3, and Caspase-9 expressions, and this was accompanied by low Bcl-2 protein expression, suggesting a major role for the activation of the intrinsic apoptosis pathway. EPS has been previously shown to exert its protective effects against ROS-mediated neurotoxicity by reducing ROS levels [23]. In this study, BZ significantly increased the ROS level and induces apoptosis in BMMNCs. However, EPS treatment was observed to protect bone marrow cells from apoptosis-induced hematotoxicity by reducing the extent of apoptosis of BMMNCs in our BIAA model. Our results also indicated that treatment with EPS was associated with the downregulation of BAX, Caspase-3, and Caspase-9 expressions and the upregulation of Bcl-2. These results suggested that the potential protective properties of EPS on BZ-induced hematotoxicity in BIAA mice may be through the inhibition of ROS-mediated oxidative stress and apoptosis.

Clinical and laboratory data from BZ-exposed workers suggested that the immune system, especially T lymphocyte subsets, plays an important role in the pathogenesis of BZ-induced hematological and immunological toxicity [16, 44]. Exposure to BZ can cause an immunosuppressive effect through the reduction of T lymphocytes such as CD4^+^ T lymphocytes [44, 45]. A decrease in CD86^+^ T lymphocytes is closely related to immune surveillance injury in gasoline station attendants exposed to airborne BZ [14]. A recent study showed that EPS can enhance immunomodulatory activities and inhibit the tumor growth in LLC-bearing mice by regulating host immune system function [22]. In our study, a significant decrease in CD3^+^, CD4^+^, CD8^+^, CD80^+^, and CD86^+^ T-cell counts and CD4^+^/CD8^+^ ratio was observed in peripheral blood in our BIAA model. However, EPS treatment reduced BZ-induced T lymphocytes injury by increasing CD3^+^ and CD4^+^ T-cell counts and CD4^+^/CD8^+^ ratio. CD80^+^ and CD86^+^ are membrane differentiation antigens and are expressed on activated human and mice peripheral blood T lymphocytes and monocytes [46, 47]. The lower levels of CD80^+^ and CD86^+^ expressions in peripheral blood lymphocytes in our BIAA model were in agreement with a report study which showed that BZ-induced immunotoxicity could be due to lower levels of CD80^+^ and CD86^+^ [14]. Although no significant difference was observed in CD80^+^ and CD86^+^ lymphocytes between the EPS pretreatment groups and the BIAA group, an increasing trend of CD80^+^ and CD86^+^ lymphocytes was observed with increasing EPS dosage. Our results demonstrated that EPS could increase CD3^+^ and CD4^+^ T-cell counts and attenuated BZ-induced immune impairment of lymphocytes. This finding is in agreement with a previous study showing that EPS has an immunomodulatory potential [20].

## 5. Conclusions

Our study showed that BZ exposure of mice for 8 weeks can cause BIAA possibly due to the ROS production by BZ exposure and consequent oxidative stress and increased apoptosis rate in BMMNCs, and a decrease in T lymphocytes in peripheral blood. Our results showed that EPS recovered the BZ-induced abnormalities in whole blood cells, immunotoxicity via regulating the T lymphocytes subpopulations, inhibited oxidative stress, and apoptosis of BMMNCs. The protective effect of EPS against BZ-induced hematotoxicity could also be due to its antioxidative, immune-modulatory, and antiapoptotic properties. These results suggested the potential therapeutic effects of EPS for the treatment of hematopoietic disorders by BZ exposure. People potentially exposed to BZ include not only occupational but also general populations, leading to BZ-induced hematotoxicity being a global public health concern. Therefore, our results will be helpful in the prevention and treatment of BZ-induced hematotoxicity and consequent BIAA. Further studies are required to uncover the more functional effects and regulatory mechanisms of EPS.

## Figures and Tables

**Table 1:** Animal grouping and treatment scheme.

Control group	BZ group	EPS group		
		Low-dose (LE)	Middle-dose (ME)	High-dose (HE)
Corn oil	BZ-880 mg/kg	EPS-20 mg/kg + BZ	EPS-100 mg/kg + BZ	EPS-200 mg/kg + BZ

## Data Availability

The table and figure data used to support the findings of this study are included in the article. All the authors declare that all the data in the manuscript were obtained by our experiments and the data are true and effective in the manuscript.

## Additional Points

- Benzene can cause oxidative stress, hematotoxicity, and immunotoxicity in male CD-1 mice.
- Epimedium polysaccharide had a dose-dependent protective effect against benzene-induced pancytopenia and loss of nucleated cells.
- Epimedium polysaccharide attenuated benzene-induced excessive reactive oxygen species and apoptosis rate in bone marrow mononuclear cells.
- Epimedium polysaccharide improved benzene-induced immune dysfunction by increasing CD3^+^ and CD4^+^ T-cell counts.
